# Nrf2 Regulates Anti-Inflammatory *A20* Deubiquitinase Induction by LPS in Macrophages in Contextual Manner

**DOI:** 10.3390/antiox10060847

**Published:** 2021-05-26

**Authors:** Haranatha R. Potteti, Lalith K. Venkareddy, Patrick M. Noone, Aparna Ankireddy, Chandramohan R. Tamatam, Dolly Mehta, Chinnaswamy Tiruppathi, Sekhar P. Reddy

**Affiliations:** 1 Departments of Pediatrics, University of Illinois, Chicago, IL 60612, USA; phreddy@uic.edu (H.R.P.); lalith15@uic.edu (L.K.V.); pnoone2@uic.edu (P.M.N.); aparna1@uic.edu (A.A.); tamatam@uic.edu (C.R.T.); 2 Department of Pharmacology, University of Illinois, Chicago, IL 60612, USA; dmehta@uic.edu (D.M.); tiruc@uic.edu (C.T.); 3 Department of Pathology, University of Illinois, Chicago, IL 60612, USA; 4 University of Illinois Cancer Center, University of Illinois, Chicago, IL 60612, USA

**Keywords:** Inflammation, deubiquitinase, macrophages, endotoxin, lung

## Abstract

The aberrant regulation of inflammatory gene transcription following oxidant and inflammatory stimuli can culminate in unchecked systemic inflammation leading to organ dysfunction. The Nrf2 transcription factor dampens cellular stress and controls inflammation by upregulating antioxidant gene expression and TNFα-induced Protein 3 (TNFAIP3, aka A20) deubiquitinase by controlling NF-kB signaling dampens tissue inflammation. Here, we report that Nrf2 is required for *A20* induction by inflammatory stimuli LPS in monocyte/bone marrow derived macrophages (MDMΦs) but not in lung-macrophages (LDMΦs). LPS-induced A20 expression was significantly lower in *Nrf2*^−/−^ MDMΦs and was not restored by antioxidant supplementation. Nrf2 deficiency markedly impaired LPS-stimulated *A20* mRNA expression *Nrf2*^−/−^ MDMΦs and ChIP assays showed Nrf2 enrichment at the promoter *Nrf2*^−/−^ MDMΦs upon LPS stimulation, demonstrating that Nrf2 directly regulates *A20* expression. Contrary to MDMΦs, LPS-stimulated *A20* expression was not largely impaired in *Nrf2*^−/−^ LDMΦs ex vivo and in vivo and ChIP assays showed lack of increased Nrf2 binding at the *A20* promoter in LDMΦ following LPS treatment. Collectively, these results demonstrate a crucial role for Nrf2 in optimal *A20* transcriptional induction in macrophages by endotoxin, and this regulation occurs in a contextual manner.

## 1. Introduction

A counterbalance between pro- and anti-inflammatory gene transcription is crucial for normal homeostasis after septic shock. Aberrant regulation of this balance culminates in unchecked systemic inflammation, leading to lung tissue damage and edema, respiratory failure, and ultimately death. However, the exact mechanisms underlying pathological inflammation leading to impaired resolution of lung injury and inflammation following septic and non-septic injuries are poorly understood. Thus, there are limited strategies, if any, that can accelerate tissue injury resolution in the clinical setting. TNFAIP3 (aka A20) is a deubiquitinase and crucial endogenous inhibitor of tissue inflammation [1,2,3] This enzyme terminates NF-κB and MAP kinase signaling by removing K-63-linked poly-ubiquitin chains on TRAF2, TRAF6, and NEMO/IKKγ, which are essential for IKK activation [4,5]. *A20* polymorphisms are associated with several autoimmune diseases [6,7,8], and *A20*-deficient (null) mice develop spontaneous inflammation and cachexia and die prematurely [9]. Furthermore, *A20* haplo-insufficiency promotes heightened inflammation in mice [9] and leads to an early onset of autoimmune disease in humans [10]. *A20* is known to be transcriptionally activated by NF-κB in response to TNFα or LPS treatment [11,12]. However, increased NF-κB levels persist under inflammatory conditions, suggesting A20 expression and/or functional activity is sub-optimal and leads to insufficient termination of NF-κB signaling. Thus, delineating the exact mechanisms underlying optimal *A20* transcription may provide novel insights into heightened inflammation caused by microbial and nonmicrobial insults.

Nuclear factor erythroid 2-like 2 (NFE2-L2 or aka Nrf2) is a transcription factor that activates gene expression required for cytoprotection, tissue regeneration, and host defense through the antioxidant response element (ARE) [13,14]. Kelch-like ECH-associated Protein 1 (Keap1) is the endogenous negative regulator of Nrf2. It retains the latter in the cytosol and enables its proteasomal degradation by the Cul3-ubiquitin ligase system, thereby limiting Nrf2-regulated gene transcription. Stressful stimuli causing oxidative or electrophilic modifications of cysteine residues in Keap1 impede Nrf2 degradation and foster its subsequent nuclear accumulation, leading to enhanced cytoprotective gene expression [13,14]. We have previously shown that Nrf2 confers protection against infectious (e.g., endotoxin) and oxidant stress (e.g., hyperoxia and mechanical ventilation) induced lung inflammatory injury [15,16,17]. Impaired resolution of lung inflammation in *Nrf2*-deficient mice is observed after oxidant-induced acute lung injury [18]. Nrf2 negatively modulates LPS-stimulated NF-κB activity and thereby suppresses inflammation during septic shock [15]. We tested whether or not Nrf2 controls the inflammatory response by modulating A20 expression levels. Here, we report that Nrf2 positively regulates *A20* expression in response to pro-inflammatory stimuli (LPS and TNFα), and that it directly binds to the endogenous *A20* promoter encompassing ARE. These results demonstrate a crucial role for Nrf2 in the regulation of optimal *A20* transcriptional induction by inflammatory stimuli and that this regulation is distinctly modulated in monocyte-derived and lung tissue-resident macrophages.

## 2. Materials and Methods

### 2.1. Mice, LPS Exposure, and Lung Cell Isolation

Wildtype (C57BL6, WT) and *Nrf2^−/−^* mice (8–16 wk males) under anesthesia were instilled intratracheally with sterile PBS (vehicle) or 10 µg of LPS (L4005; Sigma-Aldrich, St Louis, MO, USA). Mice were killed after 6 h and lung derived (resident) macrophages (LDMΦs) were isolated as previously described [19,20]. Briefly, mice were euthanized, sacrificed and lungs were instilled with 1 mL of RPMI-1640 medium containing dispase solution (Roche Applied Science, Penzberg, Germany) (0.8 U/mL) and collagenase type II (1 mg/mL) (Thermo-Fisher Scientific, Waltham, MA, USA), and incubated at 37 °C for 30 min. Lung tissue was gently teased in DMEM with 10% FBS supplemented with HEPES and antibiotics, filtered, plated on 6-well plates, and incubated at 37 °C for 30 min. Supernatant was removed, and attached macrophages were lysed with TRIzol.

For ex vivo studies, LDMΦs isolated from naïve wildtype (*Nrf2*^+/+^) and *Nrf2^−/−^* mice (8–16 weeks, males and females) separately as above and then incubated in RPMI medium with 1% FBS for at least 1 h prior to treatment with 100 ng/mL LPS (L2637; Sigma-Aldrich) or 10 ng/mL TNFα (410-MT, Biotech/R&D Systems, Minneapolis, MN, USA) and then lysed for RNA isolation. Experiments with mice were performed according to the protocol guidelines approved by the animal care and use committee at University of Illinois at Chicago.

### 2.2. Cell Culture

BMDMΦs were isolated from *WT* and *Nrf2^−/−^* mice (8–16 weeks, males and females) separately and cultured in RPMI medium supplemented with 15% L929 medium in petri-dishes for 5–6 days using standard protocol. One day prior to treatment, cells were lifted and plated on 6-well culture plates for at least 16–18 h. Cells cultured in medium with 1% FBS for 2 h and then treated with 100 ng/mL LPS or 10 ng/mL TNFα. MH-S (a murine alveolar macrophage cell line) were cultured in RPMI medium with 10% FBS and antibiotics. After 2 h serum starvation (1% FBS), MH-S were either treated with vehicle (PBS) or LPS (100 ng/mL). Mouse embryonic fibroblasts (MEFs) derived from wildtype and *Nrf2*^−/−^ mice were cultured in Iscove’s modified Dulbecco’s medium with 10% FBS and antibiotics as detailed elsewhere [21]. MEFs were cultured in medium with 1% FBS for 2 h prior to treatment with LPS (500 ng/mL) or TNFα (10 ng/mL).

### 2.3. Chromatin Immunoprecipitation (ChIP) Assays

ChIP and qPCR analysis were performed using an EZ-ChIP assay kit (17-295, EMD Millipore, Burlington, VT, USA). Briefly, BMDMΦs or MH-S cells treated with LPS were crosslinked with formaldehyde, and chromatin was prepared according the protocol. Diluted and pre-cleared chromatin was incubated with Nrf2 antibodies (SC-365949, Santa Cruz Biotechnology, Inc., Dallas, TX, USA), IgG or RNA Pol II antibodies (17-295, EMD Millipore) for 18 h at 4 °C with rotation. Samples were incubated with protein A-agarose, washed and bound DNA was eluted. Immunoprecipitated DNA was amplified by qPCR using murine *A20* promoter (Accession KX869907) specific primers: Forward, 5′-CTATTTGCTGCCTTGTAGCATC-3′ and Reverse, 5′-GTTTGTCAGTGATCCAAGTTTGT-3.’

### 2.4. Quantitative Real-Time PCR

RNA was isolated using the TRIzol reagent (Thermo Fisher). cDNA was prepared using qScript cDNA Synthesis Kit (Quanta Biosciences, Beverly Hills, CA, USA) and fast SYBR green quantitative real-time PCR (qRT-PCR) assay (Thermo Fisher) was performed using primers: *A20* (*Tnfaip3 NM_009397*, F: CAGTGGGAAGGGACACAACT, R: GCAGTGGCAGAAACTTCCTC), *Tnfaip2* (NM_009396, F: AGGAGGAGTCTGCGAAGAAGA, R: GGCAGTGGACCATCTAACTCG), *Tnfaip1 (NM_0011593920,* F: GCTGGCAA-CAAGTACGTG, R: GTGCTTTCCACATCGGTCTATG), Nqo1 (NM_008706.5, F: TTCTCTGGCCGATTCAGAGT, R: GGCTGCTTGGAGCAAAATAG), IL-1β (NM_008361.4, F: GCCTTGGGCCTCAAAGGAAAGAATC; R: GGAAGACACAGATTCCATGGTGAAG) and β-actin (NM_007393.5, F: GCAAGCAGGAGTACGATGAGT, R: AACGCAGCTCAGTAACAGTC). β-actin was used as reference to normalize and calculate the expression levels of the target genes. Relative fold change was calculated used WT control group or PBS treated cells.

### 2.5. Immunoblotting

Cells were homogenized in RIPA buffer (R0278, Sigma, St. Louis, MI, USA) with protease cocktail inhibitor (P8340, Sigma), and extract (~40 µg) was separated, blotted, and probed with A20 antibodies (C4625, Cell Signaling Technologies, Danvers, MA, USA) and β-actin antibodies (A5441, Sigma Aldrich). Immunoblots were developed using the HyGlo ECL kit (E2400, Denville Scientific Inc., Metuchen, NJ, USA) and visualized by Bio-Rad Gel Doc system.

### 2.6. Statistical Analysis

Two-way ANOVA with Tukey’s multiple comparisons test using GraphPad was used to calculate the significance between *WT* and *Nrf2*^−/−^ genotypes. Otherwise, Student’s t-test was used for comparisons with PBS controls. Error bars represent SD. *p* <0.05 are considered as significant.

## 3. Results

### 3.1. Nrf2-Deficiency Impairs LPS-Induced A20 Expression in Monocyte-Derived Macrophages

**Figure 1:**
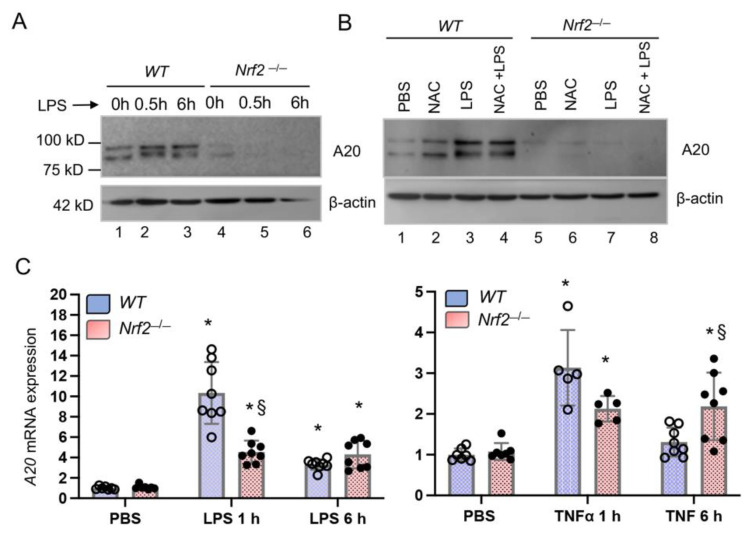
LPS-induced *A20* expression is lower in Nrf2-deficient BMDMΦs. (**A**) Cell extracts were isolated from wildtype (WT) and Nrf2^−/−^ bone marrow-derived macrophages (BMDMΦs) treated with LPS (100 ng/mL), blotted and probed with A20 antibodies. (**B**) WT and Nrf2^−/−^ BMDMΦs were treated with 5 mM N-acetylcysteine (NAC) for 16 h and then treated with LPS for 1 h. Cell extracts were probed A20 antibodies. Note that blotted membranes were cut at ~50 kD and top and bottom portion of the blots were probed with A20 and β-actin antibodies, respectively. (**C**) WT and Nrf2^−/−^ BMDMΦs isolated from male and female mice in separate experiments were treated with LPS (100 ng/mL) or TNFα (10 ng/mL) for 1 h or 6 h. Total RNA was isolated for *A20* mRNA expression analysis. Values of PBS controls used in right panel are the same as those in left panel. * versus PBS; § versus WT counterparts. LPS and TNFα stimulated *A20* mRNA expression levels in BMDMΦs of male and female mice are shown in Figure S1.

To evaluate whether Nrf2 regulates A20 expression, BMDMΦs from wildtype (*WT*) and *Nrf2*^−/−^ mice (males) were isolated and treated with PBS (control, 0 h), or with LPS for 0.5 h or 6 h. Protein extracts were isolated and A20 levels were analyzed by immunoblot analysis. As shown in Figure 1A, LPS stimulated A20 expression by 0.5 h compared to control (untreated) cells. In *Nrf2*^−/−^ BMDMΦs, basal level A20 expression is low compared to wildtype cells and was not induced by LPS, demonstrating that Nrf2 regulates A20 expression. To determine whether antioxidant supplementation to *Nrf2*^−/−^ cells will restore A20 induction by LPS, both wildtype and *Nrf2*^−/−^ BMDMΦs were cultured overnight with N-acetylcysteine (NAC) and then treated with LPS for 1 h. Cells were lysed and A20 expression was analyzed by immunoblot analysis (Figure 1B). Both basal and LPS-stimulated A20 expression were not restored in *Nrf2*^−/−^ BMDMΦs supplemented with NAC. Likewise, proteasomal inhibition did not restore LPS-stimulated A20 expression levels in *Nrf2*^−/−^ cells following LPS stimulation (data not shown). These results suggest that Nrf2 regulates *A20* expression directly at the transcriptional level. To examine whether Nrf2 directly regulates *A20* transcription, BMDMΦs isolated from *WT* and *Nrf2*^−/−^ mice of both genders (males and females) separately were treated with PBS (control, 0 h), or with LPS for 1 h or 6 h. RNA was isolated and *A20* mRNA levels were analyzed by qRT-PCR. As shown in Figure 1C, LPS strongly stimulated *A20* expression by 1 h and expression remained high above basal level up to 6 h compared to control. In *Nrf2*^−/−^ BMDMΦs, LPS-stimulated *A20* mRNA levels were significantly lower than in *WT* counterparts.

We examined whether Nrf2 deficiency broadly affects *A20* induction by inflammatory stimuli or if these effects were limited to LPS. To evaluate this aspect, *WT* and *Nrf2*^−/−^ BMDMΦs isolated above were treated with TNFα for 1 h or 3 h, and *A20* expression was analyzed by qRT-PCR. A20 was originally identified as a TNFα-inducible gene (TNFAIP3)**.** As anticipated, TNFα stimulated *A20* expression in WT BMDMΦs by 1 h, but the magnitude of induction was lower in *Nrf2*^−/−^ cells (Figure 1C). However, TNFα stimulated *A20* expression was significantly elevated in *Nrf2*^−/−^ BMDMΦs by 6 h compared to *WT* counterparts. Note that BMDMΦs isolated from both males and females showed a similar response in the context of LPS- and TNFα-stimulated *A20* expression regulation by Nrf2 (see Supplementary Figure S2).

### 3.2. A20 Induction by TNFα in Embryonic Fibroblasts is Regulated by Nrf2

**Figure 2:**
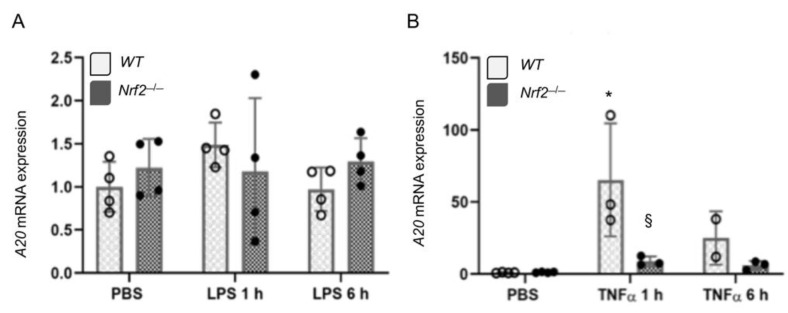
Nrf2 deficiency impairs TNFα stimulated A20 induction in embryonic fibroblasts. Wildtype (WT) MEFS and *Nrf2*^−/−^ MEFs were treated with 500 ng/mL LPS (**A**) or 10 ng/mL TNFα (**B**), RNA isolated and *A20* mRNA expression analyzed. * versus PBS; § versus WT counterparts. Values of PBS controls are from panel A as TNFα and LPS treatments were carried out in parallel.

Next we examined *A20* expression regulation by Nrf2 in another cell type, using embroyonic fibroblasts derived from *WT* and *Nrf2*^−/−^ mice. Mouse embryonic fibroblasts (MEFs) were treated with LPS or TNFα for 1 h or 6 h, RNA was isolated, and *A20* expression was analyzed by qRT-PCR. As shown in Figure 2A, LPS did not significantly stimulate *A20* expression in *WT* and *Nrf2*^−/−^ cells. Lack of LPS induced *A20* mRNA expression was anticipated as these cells compared to macrophages are known to respond poorly to LPS. However, TNFα-strongly stimulated *A20* expression, which was markedly lower in *Nrf2*^−/−^ MEFs than in *WT* counterparts (Figure 2B). Basal *A20* expression levels were not affected by Nrf2-deficiency (Figure 2A,B). These results demonstrate that Nrf2 regulates *A20* expression in multiple cell types in response to pro-inflammatory stimuli.

### 3.3. Nrf2 Does Not Regulates LPS- and TNFα-Stimulated A20 Expression in Lung Macrophages Ex Vivo

**Figure 3:**
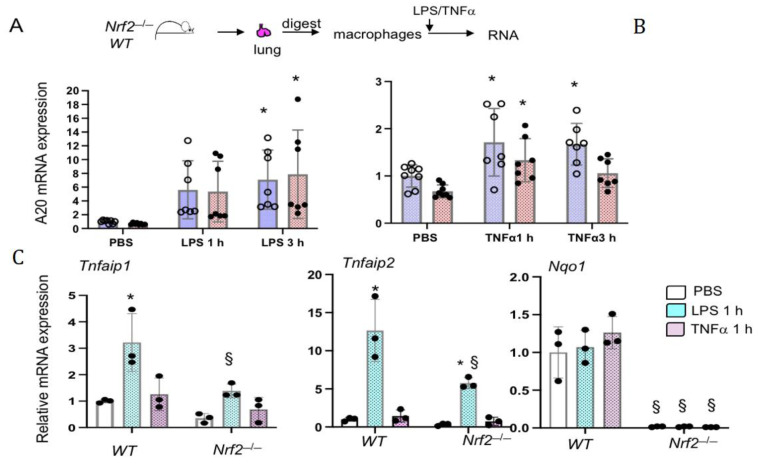
Nrf2 does not regulate LPS-stimulated *A20* transcriptional induction in LDMΦs ex vivo. Lungs from wildtype (WT) and *Nrf2*^−/−^ mice (males and females, 2-3 mice) were harvested and digested separately for macrophage isolation as outlined in schema (see methods for details). Lung-derived macrophages (LDMΦs) were treated with 100 ng/mL LPS (**A**) or 10 ng/ml TNFα (**B**) for 1 h or 3 h. Values of PBS controls used are the same as those used in panel A, as TNFα was used along with LPS treatment. LPS and TNFα stimulated *A20* expression levels in BMDMΦs of male and female mice are shown in Figure S2. (**C**) *Tnfaip1*, *Tnfaip2* and *Nqo1* mRNA expression in LDMΦs isolated from male mice treated with LPS and TNFα ex vivo for 1 h is shown. * versus PBS; § versus WT counterparts.

Previous studies have shown distinct regulation of inflammatory cytokine gene expression in lung resident macrophages compared to infiltrated macrophages (see discussion). We therefore examined *A20* expression regulation by LPS and TNFα in lung derived macrophages (LDMΦs). The lungs from naïve *WT* and *Nrf2*^−/−^ males and female mice were separately digested and macrophages were isolated as detailed in methods. We used this method mainly to minimize perturbations of lung macrophages and to mimic their naïve state in vivo [19,20]. LDMΦs were treated with LPS or TNFα for 1 h or 3 h, RNA was isolated and *A20* mRNA expression was analyzed by qRT-PCR. As shown in Figure 3, LPS-stimulated *A20* expression between *WT* and *Nrf2*^−/−^ cells was comparable (Figure 3A). Additionally, TNFα-stimulated *A20* expression was modestly lower in *Nrf2*^−/−^ LDMΦs than in *WT* counterparts (Figure 3B). LDMΦs isolated from both males and females showed a similar response in the context of *A20* expression regulation by LPS and TNFα (Supplementary Figure S3). Note that, due to variability of the magnitude of *A20* induction by LPS between LDMΦs of males and females, combined values do not show significance at the 1 h time point, contrary to their individual comparison (Supplementary Figure S3).

We next analyzed the expression levels of *Tnfaip1* (BCR E3-ubiquitin ligase complex) and *Tnfaip2* in LDMΦs isolated from male mice treated with LPS or TNFα for 1 h (Figure 3C). We found that both of them were strongly induced by LPS in *WT* cells, but their induction was impaired in *Nrf2*^−/−^ cells. In contrast to LPS, both *Tnfaip1* and *Tnfaip2* expression was not induced by TNFα. To verify that Nrf2 is functionally active in LDMΦs, we have quantified the levels of Nrf2 putative target *Nqo1* (Figure 3C). As anticipated, *Nqo1* expression was markedly lower in *Nrf2*^−/−^ LDMΦs, and its expression in *WT* (*Nrf2*^+/+^) cells was not altered by LPS or TNFα. These results suggest that Nrf2 distinctly regulates *Tnfaip1, Tnfaip2* and *Tnfaip3* expression in LDMs in response to inflammatory stimuli.

### 3.4. Nrf2 Deficiency Augments LPS-Stimulated IL-1β Expression in Both BMDMΦs and LDMΦs

**Figure 4:**
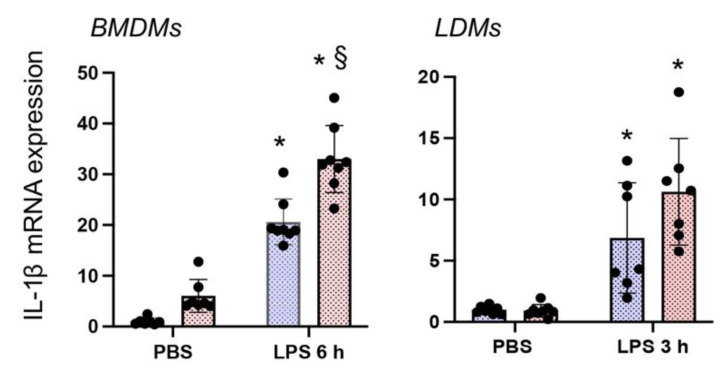
Nrf2 deficiency augments LPS-stimulated IL-1β expression in both BMDMΦs amd LDMΦs. IL-1β mRNA expression levels in BMDMΦs and LDMΦs isolated from male and female mice and treated with LPS for 6 h (BMDMΦs) and 3 h (LDMΦs), as in Figure 2 and Figure 5, was analyzed by qRT-PCR. * versus PBS; § versus WT counterparts. LPS-stimulated IL-1β mRNA levels in BMDMΦs of male and female mice are shown in Figure S3.

We next examined whether Nrf2 deficiency distinctly affects inflammatory cytokine gene expression in response to LPS in BMDMΦs and LDMΦs by measuring *IL-1β* expression levels. As shown in Figure 4, LPS-stimulated *IL-1β* expression in *Nrf2*^−/−^ BMDMΦs was greater than in *WT* counterparts. Likewise, *IL-1β* induction by LPS was more evident in *Nrf2*^−/−^ LDMΦs compared to *WT* LDMΦs. These results suggest that Nrf2 dampens LPS-stimulated *IL-1β* expression levels in both BMDMΦs and LDMΦs. In contrast to LDMΦs, however we found that *IL-1β* expression was high in *Nrf2*^−/−^ BMDMΦs compared to WT BMDMΦs under basal state.

### 3.5. Nrf2 Does Not Regulate LPS-Stimulated A20 Expression in Lung Derived Macrophages In Vivo

**Figure 5:**
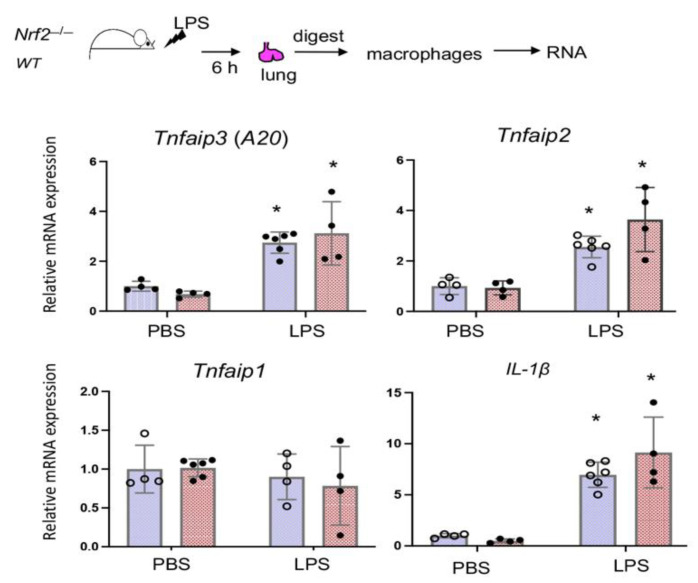
Nrf2 does not regulate *A20* induction by LPS in LDMΦs in vivo. WT and *Nrf2*^−/−^ mice (males) were treated oropharyngeally with LPS (10 µg/mouse) for 6 h, and immediately sacrificed, lungs were perfused, harvested separately from 2-3 mice and lung digest from each mouse was plated on two different culture (60 mm) dishes, and attached macrophages were used for RNA isolation and cDNA preparation. *A20* (*Tnfaip3*), *Tnfaip1*, *Tnfaip2* and *IL-1β* expression was analyzed by qRT-PCR. * versus PBS.

To examine Nrf2 regulated *A20* expression in LDMΦs in vivo, *WT* and *Nrf2*^−/−^ mice (males) treated oropharyngeally with PBS or LPS (10 µg/mouse) for 6 h. We chose this time point to determine direct effects of Nrf2 on *A20* transcriptional induction, which occurs rapidly following LPS stimulation. Moreover, we chosen this early time point to minimize the recruitment of infiltrated monocytes in the lung following LPS exposure. Mice were immediately sacrificed, lungs digested, macrophages isolated, and *A20* mRNA expression was analyzed by qRT-PCR. As shown in Figure 5, LPS-stimulated *A20* expression in LDMΦs is comparable between *WT* and *Nrf2*^−/−^ cells. *Tnfaip2* but not *Tnfaip1* expression was stimulated by LPS, but the induction was comparable between LDMΦs of two genotypes. *IL-1β* expression was more in LDMΦs of LPS treated *Nrf2*^−/−^ mice compared to *WT* counterparts, but this cytokine expression was comparable between *WT* and *Nrf2*^−/−^ mice treated with PBS.

### 3.6. Nrf2 Binds to the Endogenous A20 Promoter in BMDMΦs But Not in LDMs

**Figure 6:**
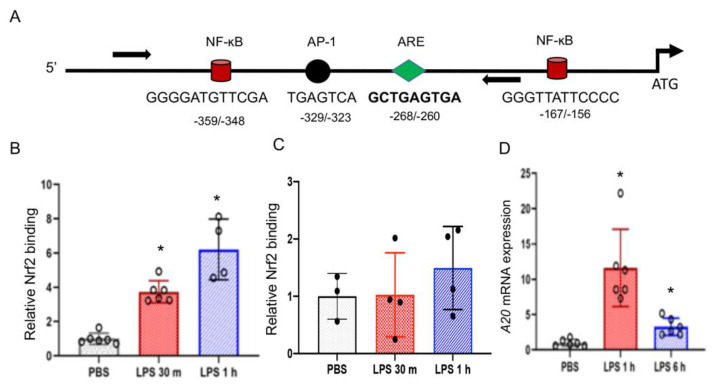
Nrf2 binding at the *A20* promoter in LPS treated BMDMΦs and LDMΦs. (**A**) Schema represents position of the antioxidant response element (ARE), and NF-κB and AP-1 binding sites of the murine *A20* promoter. BMDMs (**B**) and MH-S cells (**C**) were treated with PBS or LPS (100 ng/mL) for 30 or 60 min, crosslinked and ChIP assays were performed using Nrf2 antibodies and murine *A20* promoter specific forward and reverse primers (arrows) flanking the ARE. The relative Nrf2 binding was calculated using PBS control samples values as one arbitrary unit. (D) MH-S cells treated with LPS (100 ng/mL) for 1 h or 6 h. RNA isolated and *A20* mRNA expression was ana-lyzed. * versus PBS..

To further examine whether Nrf2 directly regulates *A20* transcription, BMDMΦs were treated with LPS for 30 min or 60 min and chromatin was cross-linked. Chromatin fragments were immunoprecipitated with anti-Nrf2 antibodies and immunoprecipitated DNA fragments were quantified using primers encompassing the Nrf2 putative binding site, ARE, located at the *A20* proximal promoter (Figure 6A). Nrf2 binding was increased at the promoter by 30 min following LPS stimulation and remained high up to 60 min (Figure 6B). RNA Pol II antibodies were used in ChIP assays to demonstrate the *A20* promoter is transcriptionally active, and IgG was used as a negative control to demonstrate specific binding (data not shown). Collectively, these results and above demonstrate that Nrf2 binds to the *A20* promoter and regulates its transcription.

To determine the nature of Nrf2 binding at the *A20* promoter in alveolar macrophages, we performed ChIP assays in a murine alveolar macrophage cell line, MH-S. We choose these cells in lieu of primary LDMs due to the requirement of large number cells for ChIP assays. MH-S cells were stimulated with LPS for different periods, chromatin was cross-linked, immunoprecipitated with Nrf2 antibodies, and ChIP-qPCR was performed as above. Contrary to BMDMΦs, Nrf2 binding in MH-S cells was modestly but not significantly increased at the promoter region by 60 min after LPS treatment (Figure 6C). To verify that *A20* mRNA expression is induced by LPS in this cell type, we have analyzed *A20* mRNA expression in LPS-treated MH-S cells. Indeed, LPS strongly stimulated *A20* mRNA expression in MH-S cells by 1 h (Figure 6D), demonstrating its inducibility in these cells. Moreover, Nrf2 expression is detectable in MH-S cells (data not shown). These results suggest Nrf2 recruitment to the *A20* promoter does not occur strongly in alveolar macrophages following LPS stimulation.

## 4. Discussion

The present study demonstrates a novel function for Nrf2 in upregulating A20 deubiquitinase expression levels in macrophages in response to inflammatory stimuli. We found that Nrf2 directly upregulates *A20* expression by binding to its promoter bearing the ARE. Furthermore, we observed that Nrf2 mediated *A20* expression in macrophages is cell- and context-specific, as evidenced by the fact that *A20* induction by LPS is Nrf2 dependent in monocyte (bone marrow)-derived macrophages but not in lung tissue resident macrophages. Previously, we have shown that deletion of *Nrf2* in mice worsens endotoxemia and sepsis, accompanied by increased levels of NF-κB activity and inflammatory cytokine gene expression in the lung [15]. A20 terminates NF-κB and MAP (JNK and p38) kinase signaling by removing K-63-linked poly-ubiquitin chains on NEMO/IKKγ, TRAF2 and TRAF6 [4,5]. Because LPS and TNFα induced *A20* transcription in macrophages is regulated by Nrf2, we propose that excessive inflammation suppression by Nrf2 is multi-faceted and occurs, at least in part, via A20 dependent termination of NF-κB and MAP kinase signaling. Concerted communication between these signaling pathways is necessary for maintaining homeostasis following microbial infection or exposure to pro-inflammatory insults.

*A20* transcriptional induction in macrophages generally peaks by 1–3 h in response to inflammatory stimuli (e.g., LPS and TNFα). Our studies performed revealed an important role for Nrf2 in the transcriptional upregulation of *A20* in macrophages in response to acute inflammatory stimuli. Both mouse and human *A20* promoters bear Nrf2 putative binding sites (i.e., AREs), and ChIP-qPCR assays showed enrichment of Nrf2 binding at the *A20* promoter in murine BMDMΦs immediately following LPS treatment, accompanied by *A20* transcription. However, our results demonstrate that Nrf2 regulated *A20* expression operates distinctly and contextually in macrophages, as we found that Nrf2 does not regulate LPS-stimulated *A20* expression in lung tissue resident macrophages. Consistent with this result, Nrf2 binding at the *A20* promoter in LPS-stimulated alveolar macrophages was not markedly increased above basal level when compared to LPS-stimulated BMDMΦs (Figure 6). ChIP-qPCR assays performed with RNA polymerase II showed enriched binding at the promoter in alveolar macrophages treated with LPS (data not shown), suggesting that lack of increased Nrf2 binding at the *A20* promoter following LPS stimulation was likely not due to lack of accessibility of chromatin to immunoprecipitation. We found reduced basal levels of *Nqo1* mRNA expression in *Nrf2*^−/−^ LDMΦs (Figure 5), verifying the lack of Nrf2 function in this cell type. LPS did not significantly stimulated *A20* expression in MEFs as they are known to be poor responders to endotoxin due to decreased expression levels of LPS receptor, TLR4, as compared to macrophages. However, TNFα stimulated *A20* expression is significantly lower in *Nrf2*^−/−^ cells compared to wildtype counterparts (Figure 2). We found that Nrf2 does not regulate TNFα-induced *A20* expression in LDMΦs but regulates expression of other genes such as *Tnfaip1* and *Tnfaip2*. Why Nrf2 does not regulate TNFα and LPS-stimulated *A20* expression in lung macrophages is unclear and warrants further studies. Differential gene expression (basal and inducible) in tissue resident macrophages and monocyte-derived macrophages have been reported [22,23,24,25,26,27]. While differential expression/activation of intracellular signaling and metabolic state could be attributed to variable transcriptional upregulation of *A20* by Nrf2, understanding distinct mechanisms underlying this process may aid in developing targeted strategies to mitigate inflammatory disorders.

Nrf2 is widely known to bind to the promoters of cytoprotective and cell survival genes and upregulate their transcription in response to oxidant stresses [28]. Mitigation of cellular stress by Nrf2 via induction of cytoprotective gene transcription is thought to play a key role in dampening excessive/chronic activation of pro-inflammatory cytokine gene expression exerted by damaged or necrotic cells in injured tissues. However, recently, it was shown that Nrf2 directly binds to the promoters of pro-inflammatory cytokines, including *IL-1β* and *IL-6* [29] and suppresses their transcriptional induction by LPS in macrophages. Nrf2 mediates this function by attenuating RNA Polymerase II enrichment at the promoters, without affecting NF-κB binding and independent of reactive oxygen species levels, which are important for its (Nrf2) stabilization. Consistent with this result, we found increased levels of *IL-1β* expression in Nrf2-deficient LDMΦs treated with LPS (Figure 4). These results suggest that Nrf2 suppresses the inflammatory response in a multi-faceted manner by directly controlling cytokine gene expression or indirectly by modulating expression of upstream negative regulators of pro-inflammatory signaling, such as A20.

Our study has certain limitations. Different culture conditions could affect macrophage cellular responses [30], as BMDMΦs are cultured for at least 5 days prior to treatment whereas LDMΦs are freshly isolated and immediately treated with LPS or TNFα. These culture conditions could, in part, explain the differences in Nrf2 mediated *A20* induction by LPS. We performed mechanistic *A20* expression regulation studies in macrophages at early time points post LPS or TNFα treatment (1–6 h), largely due to its rapid transcriptional activation and to assess Nrf2 direct effects, but not in response to chronic exposure or other inflammatory conditions. The pattern of A20 induction in BMDMs (Supplementary Figure S1) and LDMs (Supplementary Figure S2) by LPS is largely comparable between males and females. TNFα-stimulated *A20* expression was greater in female LDMs than in male counterparts, but induction pattern overall showed a similar trend. As experiments with males and females were performed on different days, the observed mRNA expression differences need to be verified by treating macrophages from both genders with LPS/TNF side by side simultaneously in order to make any definite conclusions. Macrophages exhibit greater plasticity and heterogeneity with multiple phenotypic functions during inflammatory lung injury [31,32,33,34]. It is well established that both pro- and anti-inflammatory cytokine gene expression in macrophages is regulated in a tissue-specific and spatiotemporal manner [22,23]. Thus, the exact nature of *A20* expression regulation by Nrf2 in different tissue resident and infiltrated macrophages in vivo during inflammatory lung injury and in chronic conditions require elaborative studies, including FACS and cell sorting as well as immunohistochemical analysis. The studies we have presented here can be elaborated upon to better define the nature/status of mechanisms controlling inflammatory response by Nrf2-A20 crosstalk in the context of clinical syndromes, such as ALI/ARDS, septic shock, and chronic diseases such as arthritis.

## 5. Conclusions

In summary, the present study demonstrates that Nrf2 acts as an upstream positive upregulator of anti-inflammatory cytokine signaling mediated by the A20 deubiquitinase, whose dysfunction or haplo-insufficiency leads to inflammatory disorders in mice and humans, respectively. Our results suggest that Nrf2 mediated *A20* expression regulation in macrophages in response to acute pro-inflammatory mediators occurs distinctly in a tissue and cell type specific manner (i.e., monocyte-derived versus tissue resident macrophages). Based on these results and previous observations, we propose that Nrf2 directly counterbalances the unwarranted cytokine transcriptional response induced by oxidant stresses and pro-inflammatory stimuli in a multi-faceted manner in order to mitigate amplified inflammation and maintain homeostasis.

## Data Availability

Data is contained within the article.

## Supplementary Materials

The following are available online at https://www.mdpi.com/article/10.3390/antiox10060847/s1, Figure S1, LPS and TNFα-stimulated *A20* mRNA expression in BMDMΦs isolated from *Nrf2*^−/−^ male and female mice and *WT* counterparts; Figure S2, LPS and TNFα-stimulated *A20* mRNA expression in LDMΦs isolated from *Nrf2*^−/−^ and *WT* male and female mice; Figure S3, LPS-stimulated *IL-1**β* mRNA expression in BMDMΦs and LDMΦs isolated from *Nrf2*^−/−^ male and female mice and *WT* counterparts.
