# Supplementary figures and images for: Nrf2 Regulates Anti-Inflammatory A20 Deubiquitinase Induction by LPS in Macrophages in Contextual Manner
Source: Antioxidants (Basel). 2021 May 26;10(6):847. doi: 10.3390/antiox10060847 (PMC8228212; doi:10.3390/antiox10060847)

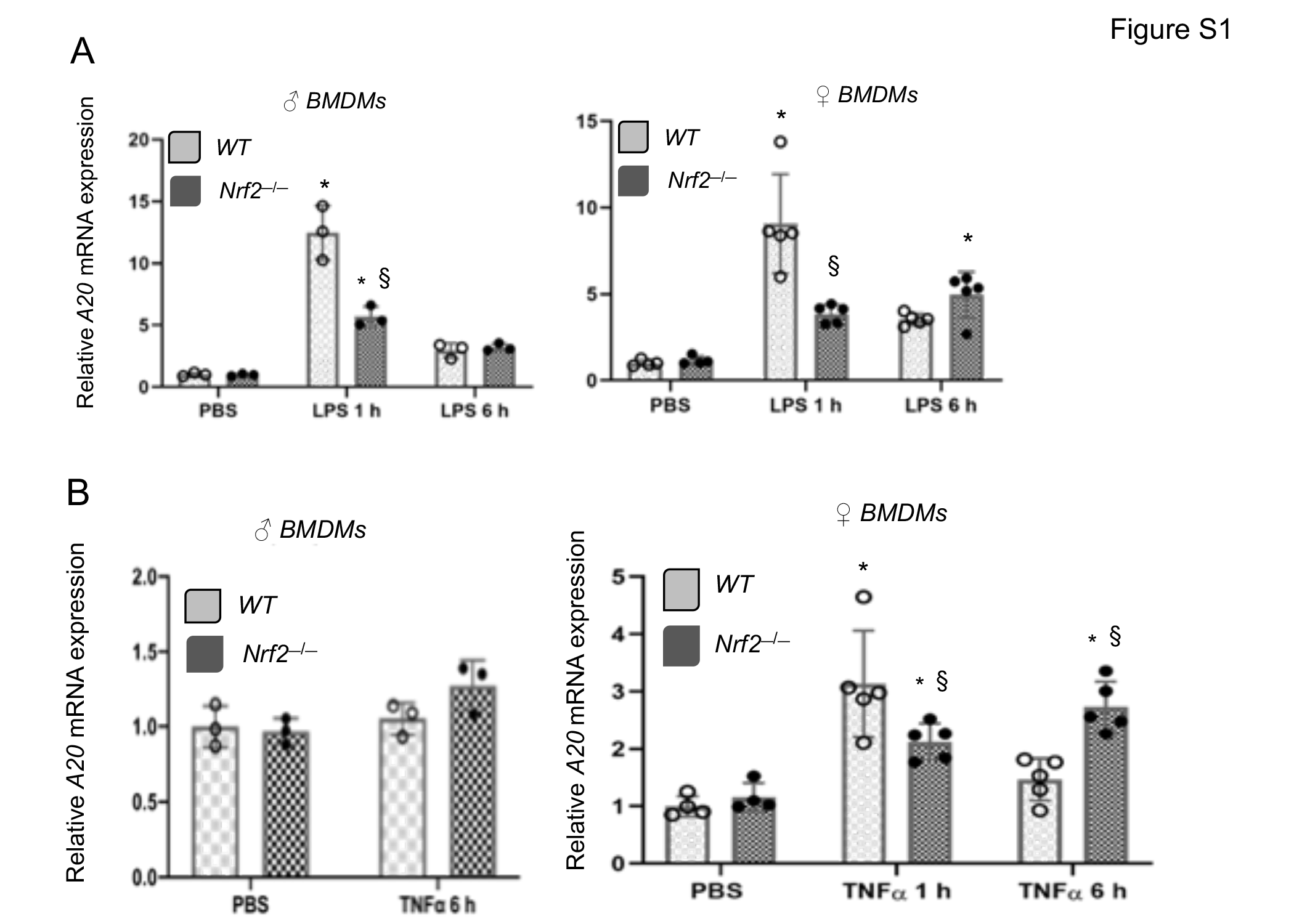

Supplement: Supplementary file 1 [file antioxidants-10-00847-s001.zip › Figure S1- antioxidants.tif]

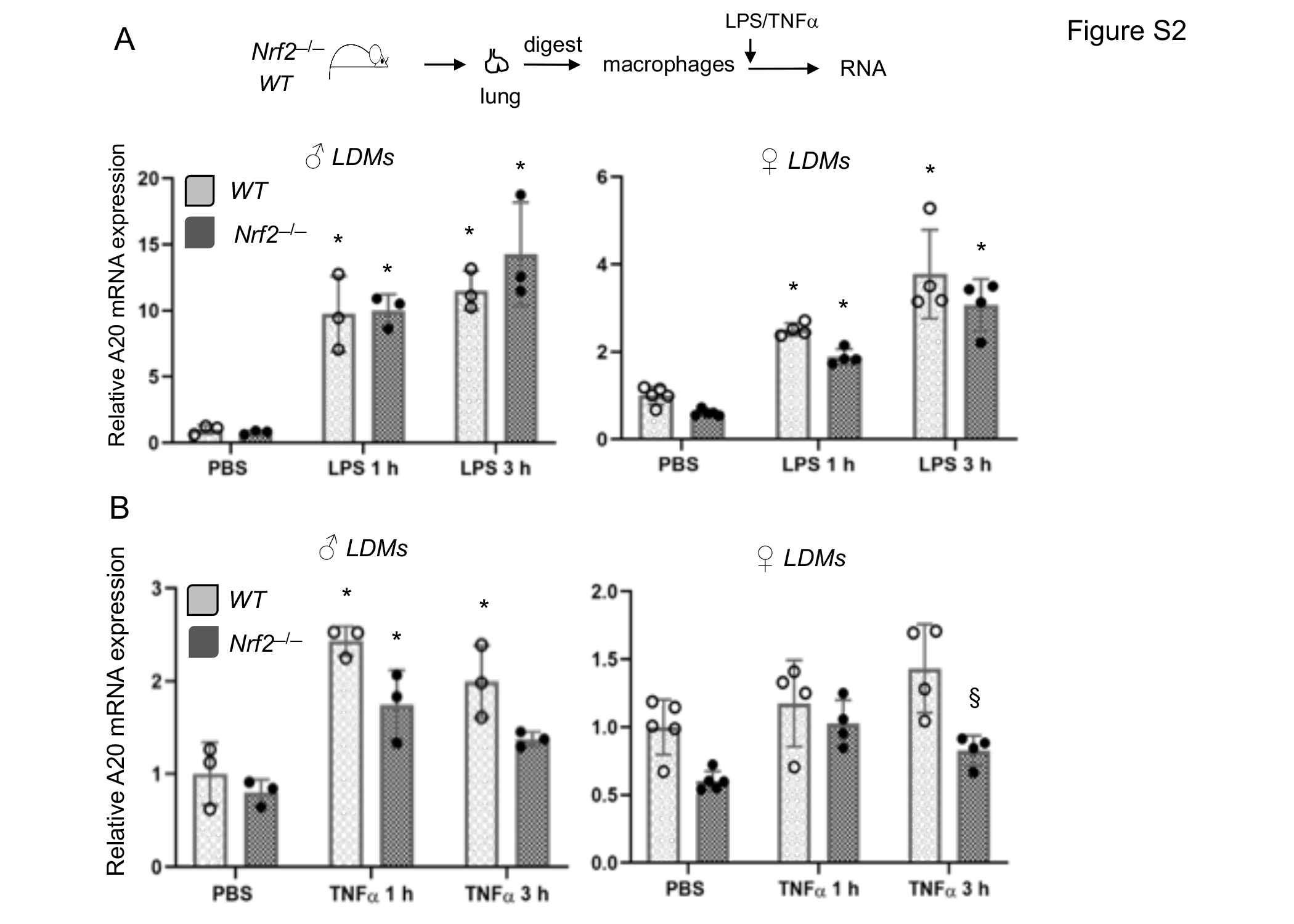

Supplement: Supplementary file 1 [file antioxidants-10-00847-s001.zip › Figure S2.antioxidants.tif]

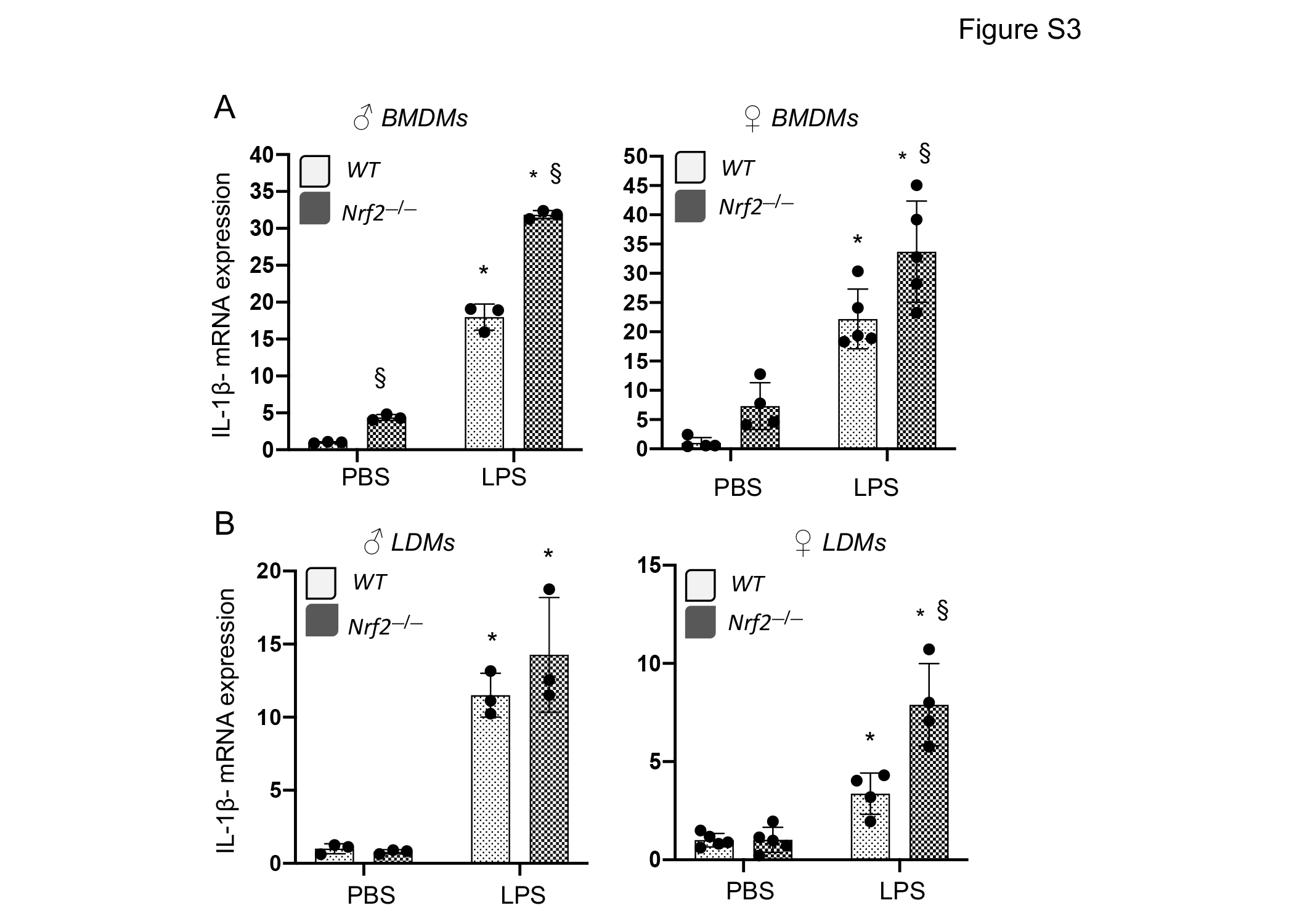

Supplement: Supplementary file 1 [file antioxidants-10-00847-s001.zip › Figure S3.antixodant.tif]
